# Mesenchymal Stem Cell-Derived Extracellular Vesicles: Challenges in Clinical Applications

**DOI:** 10.3389/fcell.2020.00149

**Published:** 2020-03-12

**Authors:** Austin Gowen, Farah Shahjin, Subhash Chand, Katherine E. Odegaard, Sowmya V. Yelamanchili

**Affiliations:** Department of Anesthesiology, University of Nebraska Medical Center, Omaha, NE, United States

**Keywords:** mesenchym stem cell, extracellular vesicle, stem cell therapeutics, biodistribution, clinical use

## Abstract

Stem cell therapy has garnered much attention and application in the past decades for the treatment of diseases and injuries. Mesenchymal stem cells (MSCs) are studied most extensively for their therapeutic roles, which appear to be derived from their paracrine activity. Recent studies suggest a critical therapeutic role for extracellular vesicles (EV) secreted by MSCs. EV are nano-sized membrane-bound vesicles that shuttle important biomolecules between cells to maintain physiological homeostasis. Studies show that EV from MSCs (MSC-EV) have regenerative and anti-inflammatory properties. The use of MSC-EV, as an alternative to MSCs, confers several advantages, such as higher safety profile, lower immunogenicity, and the ability to cross biological barriers, and avoids complications that arise from stem cell-induced ectopic tumor formation, entrapment in lung microvasculature, and immune rejection. These advantages and the growing body of evidence suggesting that MSC-EV display therapeutic roles contribute to the strong rationale for developing EV as an alternative therapeutic option. Despite the success in preclinical studies, use of MSC-EV in clinical settings will require careful consideration; specifically, several critical issues such as (i) production methods, (ii) quantification and characterization, (iii) pharmacokinetics, targeting and transfer to the target sites, and (iv) safety profile assessments need to be resolved. Keeping these issues in mind, the aim of this mini-review is to shed light on the challenges faced in MSC-EV research in translating successful preclinical studies to clinical platforms.

## Introduction

Stem cell therapy has garnered much attention and application in the past decades for the treatment of diseases and injuries. Among the different stem cell types, mesenchymal stem cells (MSCs) are studied most extensively, particularly for their application in regenerative medicine and tissue engineering (Brooke et al., 2007). MSCs are being widely researched for their therapeutic roles. MSCs are multipotent stromal cells that can differentiate into a variety of cell types (Ullah et al., 2015) and can be obtained from four common sources: adipose tissue, peripheral blood, bone marrow, and umbilical cord/blood (Gimble and Guilak, 2003; Timmins et al., 2012; Roura et al., 2015). Initially, the therapeutic efficiency of MSCs was attributed to their ability to migrate and engraft in target tissues; however, studies show that systemically administered MSCs rarely reach the target in great numbers (Gao et al., 2001), suggesting that the biological effects observed due to stem cell administration are likely due to their secreted factors. These secreted factors are attributed to several beneficial effects, such as neuroprotection, neurogenesis, myocardial protection, inflammation, etc (Lai et al., 2010; Yagi et al., 2010; Hsieh et al., 2013).

Recent studies on extracellular vesicles (EV) and their function have shed light on their crucial role in mediating cell-to-cell communication (Yáñez-Mó et al., 2015). EV are nano-sized membrane-bound vesicles (size range 30–1000 nm) that shuttle important biomolecules between cells (Valadi et al., 2007; Mittelbrunn and Sanchez-Madrid, 2012; Tetta et al., 2013), maintain physiological homeostasis (Zhang et al., 2019), and influence pathogenesis (Vakhshiteh et al., 2019). Studies show that EV from MSCs (MSC-EV) have regenerative and anti-inflammatory properties in animal models of stroke (Doeppner et al., 2015; Bang and Kim, 2019; Dabrowska et al., 2019), traumatic brain injury (Kim et al., 2016; Das et al., 2019; Ni et al., 2019), wound healing (Zhang et al., 2015), and perinatal brain injury (Thomi et al., 2019). Recent studies demonstrated that MSC-EV exert biological effects comparable to those of the parent cells (Bruno et al., 2009; Lai et al., 2010; Nawaz et al., 2016; Shao et al., 2017; Baek et al., 2019) and mediate the paracrine effects of the MSCs (Camussi et al., 2013; Xin et al., 2013a). While there are only a few studies that directly compare MSC-EV treatments to MSCs treatments, the overlapping effects seem to indicate that MSCs are merely the vehicle and that MSC-EV have a greater likelihood of impacting damaged areas (Moon et al., 2019). The use of MSC-EV, as an alternative to MSCs, confers several advantages such as higher safety profile (Yeo et al., 2013), lower immunogenicity (Natasha et al., 2014), and the ability to cross biological barriers (Zhuang et al., 2011). Additionally, use of MSC-EV avoids complications that arise from stem cell-induced ectopic tumor formation, entrapment in lung microvasculature, and immune rejection (Badillo et al., 2007; Jeong et al., 2011; Wang et al., 2015; Fennema et al., 2018). These advantages and the growing evidence of MSC-EV having therapeutic roles contribute to the strong rationale, if not the necessity, for developing EV as therapeutic treatment options.

## Preclinical Studies of Msc-Ev

**FIGURE 1:**
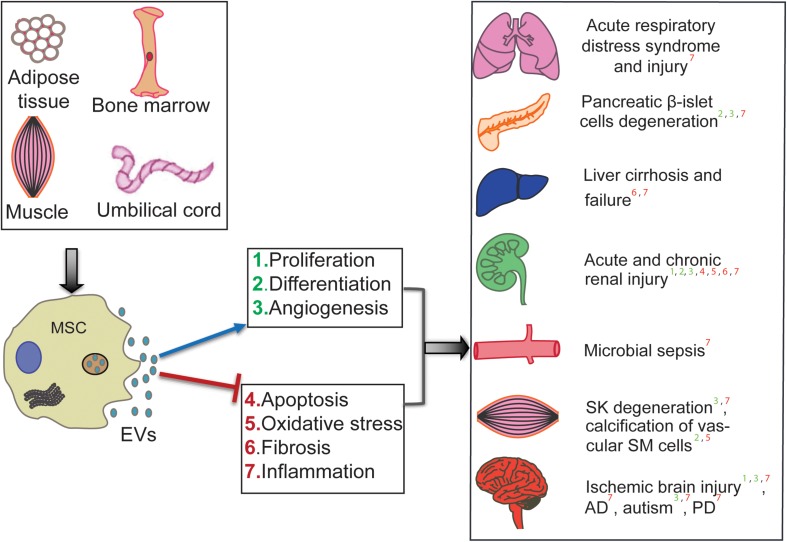
Therapeutic roles of human MSC-derived EV in various diseases. MSC-EV from different sources (hUCMSCs, hBMSCs, hATMSCs) are shown to ameliorate disease conditions such as Alzheimer’s (AD), Parkinson’s (PD), and cirrhosis. It also effects cell and organ injuries such as microbial ischemic brain injury, calcification of Smooth Muscle (SM) cells, Skeletal Muscle (SK) degeneration, chronic and acute renal injury, Pancreatic degeneration, and Acute respiratory distress/injury. The mechanism of affect is shown by positive regulation in the blue arrow and negative regulation in the red line.

The therapeutic efficiency of human MSC-EV has been tested in preclinical animal models and across many diseases and injuries. Li et al. (2013) showed that human umbilical cord MSC (hUCMSC)-derived exosomes (that are extracellular vesicles of 30–150 nm range) could ameliorate the carbon tetrachloride-induced liver fibrosis by providing hepatic protection and inhibiting the detrimental epithelial-to-mesenchymal transition. Further, hUCMSC-derived EV are shown to ameliorate experimental autoimmune uveoretinitis by inhibiting inflammatory cell migration (Bai et al., 2017). Exosomes from hUCMSCs are also suggested as therapeutic tools for cisplatin-induced nephrotoxicity (Zhou et al., 2013). Bian et al. (2014) demonstrated that hUCMSC-derived EV protect cardiac tissue from ischemic injury, partly by promoting angiogenesis, in a rat model of myocardial infarction. In atopic dermatitis mouse models, intravenous administration of EV from human adipose tissue-derived MSCs (hAMSCs) has shown anti-atopic effects (Cho et al., 2018). EV from hAMSCs are also shown to possess therapeutic potential in neurodegenerative disorders, such as Alzheimer’s disease (AD), and Huntington’s disease. In an *in vitro* AD mouse model, hAMSC-derived EV are shown to ameliorate the progression of beta-amyloid-induced neuronal death (Lee et al., 2018). Investigation of the therapeutic effects of hAMSC-derived exosomes in *in vitro* Huntington’s model revealed their neuroprotective effects; the Huntington’s disease phenotype was ameliorated via modulation of mutant Huntington aggregates’ mitochondrial and apoptotic functions (Lee et al., 2016). Interestingly, this neuroprotective property seems to influence the neurodevelopment of the fetal brain; it has been shown that application of human bone marrow (hBMMSC)-derived EV is capable of protecting the development of fetal brains afflicted with hypoxia (Ophelders et al., 2016). As highlighted in Figure 1, several preclinical studies showed success in applying MSC-EV; however, generating success in the lab is not enough to generate conclusive clinical results.

## Clinical Use of Msc-Ev

There are few published studies demonstrating the clinical effectiveness of MSC-EV. In one study, hBMMSC-EV were tested in patients suffering from steroid refractory graft-versus-host disease (Kordelas et al., 2014). Outcomes of the study revealed significant improvements in graft-versus-host disease symptoms. In another study, administration of hUCMSC-EV resulted in overall improvement in kidney function in grade III-IV chronic kidney disease patients (Nassar et al., 2016). Nassar et al. (2016) conducted a clinical trial to assess the effects of hUCMSC-EV on pancreatic islet beta cell mass in Type-1 diabetic patients (trial NCT02138331). Results of this clinical trial are not yet published. However, a supporting preclinical study suggested that intravenous administration of hUCMSC led to reduced blood sugar level as the main paracrine approach of MSCs and partially reversed insulin resistance in Type 2 diabetes mellitus indirectly to accelerate glucose metabolism (Yaoxiang et al., 2018). Also, with other MSC, it was shown that hBMMSCs led to the suppression of autoimmunity and the regeneration of islet beta cells, therefore preventing the onset of Type-1 diabetes (Ezquer et al., 2012; Zhao et al., 2012). Moreover, there are other ongoing trials conducted to determine the safety and efficacy of human MSC-EV – one led by Zhang et al. (NCT03437759) using hUCMSCs to promote healing of large and refractory macular holes and another by Zali et al. (NCT03384433) using miR-124-loaded exosomes in patients with acute ischemic stroke. While many clinical studies are in the recruitment and active phases, much of these terminate without producing a significant publication.

## Challenges in Clinical Applications

Despite the therapeutic success of MSC-EV in preclinical studies, use of these EV in clinical settings will require resolution of several critical issues such as (i) large-scale production and isolation methods, (ii) methods for rapid and accurate quantification and characterization of EV, (iii) precise content characterization of the cargo, (iv) pharmacokinetics, targeting and transfer mechanisms of EV to the target sites, and (v) safety profiles to determine the optimal clinical dosage and possible toxicities upon repeated administration.

### Large-Scale Production of MSC-EV

Traditional methods for maintenance and expansion of cells rely on a two-dimensional culture technique. Long-term passaging to produce sustainable quantities of EV may cause the cells to lose clonal and differentiation capacity (McKee and Chaudhry, 2017). Therefore, there is an urgent need for development of methods for reliable expansion of MSCs to mass produce EV for clinical use. Current methods of expansion of MSCs are labor-intensive and involve several procedures. The methods available for MSCs culture expansions are: (i) traditional tissue culture techniques in flasks (Nekanti et al., 2010; Oliver-Vila et al., 2016) or (ii) use of three-dimensional culturing bioreactors made up of polysulphone hollow fibers with semi-permeable membranes that greatly increase the surface area, as described by Mennan et al. (2019) and (McKee and Chaudhry, 2017). Unfortunately, the existing methods for EV production have low yields and are not scalable, impeding the progress of preclinical and clinical use of EV as therapeutics (Whitford and Guterstam, 2019). Large-scale EV production employs the use of large or multi-layer culture flasks, fixed-bed bioreactors, in-stirred tank bioreactors, or continuous production in perfusion reactors (Colao et al., 2018). Most of these methods aim to increase the EV production through maximizing the culture surface area as compared to the conventional planar cell culture in flasks (Whitford et al., 2015). Supporting this notion, a recent study showed that cultivation of hUCMSCs in scalable microcarrier-based three-dimensional cultures resulted in twenty-fold greater yield of EV than two-dimensional cultures (Haraszti et al., 2018).

Several technical factors when using cell culture supernatants for EV extraction need to be standardized to ensure batch-to-batch reproducibility and lot-consistent EV-production (Witwer et al., 2013). Many factors can affect the quality and quantity of the EV produced from the MSCs, such as cellular confluence, early versus later passage of cells, oxygen concentration, cytokines, heparin, and serum content of the medium (Lener et al., 2015). For instance, studies show that fetal bovine serum (FBS), a nutrient for growing cells in culture, has RNA-containing EV that can affect the cell culture behavior, highlighting the importance of developing a protocol where EV are generated without such interferences (Shelke et al., 2014). Also, serum-free cultures are shown to alter EV quantity and protein composition (Li et al., 2015). Addressing this issue, Pachler et al. (2017) developed a Good Manufacturing Practice (GMP)-grade standard protocol where they showed that hBMMSCs cultured in EV-depleted medium with reduced pooled human platelet lysate (a serum-free medium) (i) retained their morphology, phenotype, viability and differential potential, (ii) strongly affected hBMMSC proliferation and differentiation capacities, (iii) were enriched in hBMMSC-EV, and (iv) showed unchanged EV-RNA profiles that originated from hBMMSCs (Pachler et al., 2017). This study offers an option for GMP-compliant large-scale production of MSCs and MSC-EV. Apart from manipulating the culture conditions, manipulating the EV-biogenesis biology may also improve the yield of EV (Phan et al., 2018).

### Effective and Scalable EV Isolation Methods From MSCs Culture Medium

Besides large-scale manufacturing of EV, scalable EV isolation techniques are lacking, making clinical translation of EV therapeutics difficult. Currently, there are various methods available for EV isolation (Lotvall et al., 2014; Li et al., 2019); however, there is no state-of-the-art technology to isolate EV in large quantities for clinical use. In research, there are five major isolation methods: (i) differential centrifugation, (ii) density gradient ultracentrifugation, (iii) size-exclusion chromatography (SEC), (iv) precipitation, and (v) immune-based capture method (Colao et al., 2018). Interestingly, many research groups (Nordin et al., 2015; Benedikter et al., 2017; Mol et al., 2017) have demonstrated that EV isolated from stem cell culture by ultrafiltration followed by SEC results in higher yield while preserving EV biophysical and functional properties (Monguio-Tortajada et al., 2019).

The popularity of SEC in both therapeutics and biomarker discovery for disease diagnosis was explored by Stranska et al. (2018) to demonstrate the superiority of SEC qEV (Izon Science) over affinity-based EV isolation method (using exoEasy kit, Qiagen) from human plasma. Intriguingly, SEC alone is unable to separate plasma EV from lipoproteins unless combined with density gradient isolation (Karimi et al., 2018).

### Biodistribution and Targeting of MSC-EV to Target Tissues

To investigate MSC-EV as a therapeutic tool, it is critical to consider their biodistribution and targeting mechanisms *in vivo*. One method of studying different tissue targets in living animals is optical imaging (OI). This non-invasive method can visualize labeled cells *in vivo* using near-infrared (NIR) dyes that maximize depth of tissue penetration and reduce background (Rao et al., 2007; Boddington et al., 2008; Tögel et al., 2008; Grange et al., 2014; Wen et al., 2019). In a mouse model of acute kidney injury (AKI), Grange et al. (2014) used two methods for the labeling of MSC-EV: direct labeling of purified EV and generation of labeled EV from MSCs pre-incubated with NIR dye. They found that EV were detectable in whole-body images and dissected kidneys using OI, and EV that were directly labeled with NIR dye showed higher and brighter fluorescence compared to the labeled EV produced by MSCs. They also found that MSC-EV accumulated in the kidneys of their AKI mice but not in controls. MSCs are recruited to sites of injury via receptor-mediated interactions (Herrera et al., 2007), therefore MSC-EV, which have the same membrane receptors of MSCs, may be recruited to the site of injury via the same mechanism (Grange et al., 2014).

Researchers have used different dyes to track the biodistribution of EV after their administration. Wen et al. (2019) used DiD lipid dye-labeled MSC-EV to assess their distribution in mice under various conditions. The DiD-labeled MSC-EV distributed maximally in the liver and spleen, lesser in the bone marrow of the spine, femur, and tibia, and were undetectable in the lung, heart, and kidney (Wen et al., 2019). PKH-26A, a lipophilic dye that integrates into cell membranes, is commonly used to label MSC-EV (Bucan et al., 2019; Karaoz et al., 2019; Kuang et al., 2019). Bucan et al. (2019) evaluated the effects of rat adipose-derived MSC-EV (rAMSC-EV) on sciatic nerve regeneration and neurite growth. rAMSC-EV enhanced the regeneration of the sciatic nerve *in vivo* after injury and neurite growth *in vitro*. They also characterized neural growth factor transcripts in rAMSC-EV (Bucan et al., 2019). Another study from Wang et al. (2019) used DIO to label MSC-EV in a rat carotid artery balloon injury model. They found that MSC-EV can transfer miR−125b to vascular smooth muscle cells, which can attenuate neointimal formation and could be a therapeutic target of vascular diseases (Wang et al., 2019). There are also several reports of labeling MSC-EV with different labeling agents such as DiI (1,1′-Dioctadecyl-3,3,3′,3′-Tetramethylindocarbocyanine Perchlorate), Alexa fluor 488, and gadolinium for locating the biodistribution of EV (Abello et al., 2019; Chew et al., 2019; Cui et al., 2019).

Furthermore, a study from Moon et al. (2019) investigated the biodistribution, therapeutic efficacy, and mode of action of MSC-EV in a preclinical rat model of stroke. This study used PKH26 or 5-(and-6)-carboxyfluorescein diacetate succinimidyl ester (CFSE) to label EV for *in vivo* tracking. EV were identified and counted using flow cytometry, and Nanosight nanoparticle tracking analysis was used to measure size and morphology (Moon et al., 2019). They found that MSC-EV migrated to the infarcted brain. While MSC-EV accumulated in the infarcted brain in a dose-dependent manner, injected MSCs aggregated in the lung and liver with increasing doses, again highlighting that MSCs rarely make it to target tissues (Gao et al., 2001).

The mechanism of therapeutic action of EV is still unclear. EV-cargo can include membrane proteins, cytoplasmic proteins, mRNAs, and microRNAs, which can all be delivered to recipient cells. It is speculated that the therapeutic effect of EV comes through the transfer of miRNAs to diseased and injured cells (György et al., 2011). Studies have shown that miRNAs in MSC-EV influence physiology and pathophysiology of microenvironments (Xin et al., 2013b; Moon et al., 2019). Additionally, miRNAs from MSC-EV have been shown to influence cardiac regeneration and protection (Nouraee and Mowla, 2015). Strategies for loading and modifying the EV-cargo exist; electroporation, freeze-thaw cycles, saponin-mediated loading, and hypotonic dialysis have all been studied for use in exogenously loading EV (Kotmakçı and Çetintaş, 2015; Mendt et al., 2018). EV cargo may also influence EV migration. It is speculated that MSC-EV express chemokine receptors that facilitate targeting to injured regions (Kim et al., 2012; Moon et al., 2019). Decoration of the EV surface with phosphatidylserine-binding and HER2-targeting proteins has been shown to increase EV delivery to HER2-expressing cells (Wang et al., 2018). Other studies demonstrate the feasibility of this decorating method of targeting EV to specific tissues (Alvarez-Erviti et al., 2011; Kooijmans et al., 2016; Antes et al., 2018), so perhaps these strategies may be applied to MSC-EV as well. Though the exact mechanism is not known, it is speculated that MSC-EV function similarly to MSCs. MSCs exert therapeutic effects through the secretion of factors that reduce cellular injury and promote repair, and MSC-EV may function as communication vehicles employed by MSCs to signal support from the tissue microenvironment (Katsuda and Ochiya, 2015; Yin et al., 2019).

### Safety Profile

With any therapeutic treatment, a safety profile must be established. While EV-based treatment is in its clinical infancy we know that many of the harmful effects of cell therapies are absent in EV-based treatments. The main apprehension for using stem cell therapy is the differentiation of the transplanted MSCs and the potential of MSCs to suppress anti-tumor immune responses and act as a progenitor for blood vessels, which potentially promote tumor growth and metastasis (Burrello et al., 2016). Further, MSCs are hindered by their tumorigenicity, immunogenicity, and genomic mutability (Heng et al., 2004; Klyushnenkova et al., 2005; Zhang et al., 2012). Fortunately, MSC-EV are not affected by the above-mentioned limitations. A few clinical trials have been performed utilizing EV (not derived from MSCs) and these studies have established good safety profiles for treatments with ascite- and dendrite-derived EV (Pitt et al., 2016). EV inherently lack the features to cause the above-mentioned issues which, for many researchers, mark them as attractive candidates for use as therapeutic agents. Future clinical research will likely see a huge upswing in the utilization of stem cell-derived EV in place of its progenitor cell sources.

## Conclusion

The future of MSCs has long been anticipated, and there is no doubt with the discoveries coming forth and the exponentially increasing amount of clinical trials that they will provide therapies for a myriad of heretofore untreated maladies. However, it is possible that the future of MSCs will be dominated by MSC-EV. The EV’s ability to operate similarly to MSCs while not possessing many of the drawbacks extant in cell-based therapies provide them a unique niche in therapeutics.

The future of MSC-EV is dependent on the large-scale culturing of MSCs. This key step in manufacturing will open the door for them to be considered a successful therapeutic option. Standardizing treated culturing of MSCs will be a necessary future development; these EV will be treated in hypoxic conditions and likely treated with miRNA applications. Both of these conditions will significantly increase MSC growth while also increasing release of EV. Additionally, increasing the proliferation of EV isolation is another issue. For therapeutic purposes, many isolation methods can be ignored for either damage, interference, or low yield of EV. There are increasing developments being made in the field of EV isolation and characterization. SEC, with its ability to provide unaltered and selective EV, has replaced standard methodologies like immunoprecipitation and density-gradient centrifugation. Despite EV being touted for the lack of a toxic profile, the ability to include a range of specifically sized EV is an important focus in the field of EV research, especially in therapeutics. SEC allows for the more precise extraction of EV, which can ensure specific and more efficient treatments.

The biodistribution and targeting of MSC-EV is a simpler matter, despite being poorly understood. These EV act as key messengers between MSCs and damaged tissues. While MSCs might aggregate in unintended organs and tissues, their secreted EV target damaged tissues. To increase our understanding of potentially confounding effects of MSC-EV, a greater understanding of their targeting and biodistribution is necessary. Presently, clinical and preclinical trials have not reported unintended targeted effects. With few complications and a range of benefits, MSC-EV are increasingly researched despite issues with large-scale production.

## Author Contributions

AG and FS wrote the initial draft. KO edited the manuscript. SC contributed the figure and SY edited and approved the final manuscript.

## Conflict of Interest

The authors declare that the research was conducted in the absence of any commercial or financial relationships that could be construed as a potential conflict of interest.

## References

[B1] Abello J. Nguyen T. D. T. Marasini R. Aryal S. Weiss M. L. (2019). Biodistribution of gadolinium- and near infrared-labeled human umbilical cord mesenchymal stromal cell-derived exosomes in tumor bearing mice. *Theranostics* 9 2325–2345. 10.7150/thno.30030 31149047 PMC6531310

[B2] Alvarez-Erviti L. Seow Y. Yin H. Betts C. Lakhal S. Wood M. J. (2011). Delivery of siRNA to the mouse brain by systemic injection of targeted exosomes. *Nat. Biotechnol.* 29 341–345. 10.1038/nbt.1807 21423189

[B3] Antes T. J. Middleton R. C. Luther K. M. Ijichi T. Peck K. A. Liu W. J. (2018). Targeting extracellular vesicles to injured tissue using membrane cloaking and surface display. *J. Nanobiotechnol.* 16:61. 10.1186/s12951-018-0388-4 30165851 PMC6116387

[B4] Badillo A. T. Beggs K. J. Javazon E. H. Tebbets J. C. Flake A. W. (2007). Murine bone marrow stromal progenitor cells elicit an in vivo cellular and humoral alloimmune response. *Biol. Blood Marrow Transplant.* 13 412–422. 10.1016/j.bbmt.2006.12.447 17382248 PMC1892590

[B5] Baek G. Choi H. Kim Y. Lee H.-C. Choi C. (2019). Mesenchymal stem cell-derived extracellular vesicles as therapeutics and as a drug delivery platform. *Stem Cells Transl. Med.* 8 880–886. 10.1002/sctm.18-0226 31045328 PMC6708072

[B6] Bai L. Shao H. Wang H. Zhang Z. Su C. Dong L. (2017). Effects of mesenchymal stem cell-derived exosomes on experimental autoimmune uveitis. *Sci. Rep.* 7:4323. 10.1038/s41598-017-04559-y 28659587 PMC5489510

[B7] Bang O. Y. Kim E. H. (2019). Mesenchymal stem cell-derived extracellular vesicle therapy for stroke: challenges and progress. *Front. Neurol.* 10:211. 10.3389/fneur.2019.00211 30915025 PMC6422999

[B8] Benedikter B. J. Bouwman F. G. Vajen T. Heinzmann A. C. A. Grauls G. Mariman E. C. (2017). Ultrafiltration combined with size exclusion chromatography efficiently isolates extracellular vesicles from cell culture media for compositional and functional studies. *Sci. Rep.* 7:15297. 10.1038/s41598-017-15717-7 29127410 PMC5681555

[B9] Bian S. Zhang L. Duan L. Wang X. Min Y. Yu H. (2014). Extracellular vesicles derived from human bone marrow mesenchymal stem cells promote angiogenesis in a rat myocardial infarction model. *J. Mol. Med. (Berl)* 92 387–397. 10.1007/s00109-013-1110-5 24337504

[B10] Boddington S. Henning T. D. Sutton E. J. Daldrup-Link H. E. (2008). Labeling stem cells with fluorescent dyes for non-invasive detection with optical imaging. *J. Vis. Exp.* 14:686. 10.3791/686 19066580 PMC2582850

[B11] Brooke G. Cook M. Blair C. Han R. Heazlewood C. Jones B. (2007). Therapeutic applications of mesenchymal stromal cells. *Semin. Cell Dev. Biol.* 18 846–858. 10.1016/j.semcdb.2007.09.012 18024097

[B12] Bruno S. Grange C. Deregibus M. C. Calogero R. A. Saviozzi S. Collino F. (2009). Mesenchymal stem cell-derived microvesicles protect against acute tubular injury. *J. Am. Soc. Nephrol.* 20 1053–1067. 10.1681/asn.2008070798 19389847 PMC2676194

[B13] Bucan V. Vaslaitis D. Peck C. T. Strauss S. Vogt P. M. Radtke C. (2019). Effect of exosomes from rat adipose-derived mesenchymal stem cells on neurite outgrowth and sciatic nerve regeneration after crush injury. *Mol. Neurobiol.* 56 1812–1824. 10.1007/s12035-018-1172-z 29931510 PMC6394792

[B14] Burrello J. Monticone S. Gai C. Gomez Y. Kholia S. Camussi G. (2016). Stem cell-derived extracellular vesicles and immune-modulation. *Front. Cell Dev. Biol.* 4:83 10.3389/fcell.2016.00083 PMC4992732 27597941

[B15] Camussi G. Deregibus M. C. Cantaluppi V. (2013). Role of stem-cell-derived microvesicles in the paracrine action of stem cells. *Biochem. Soc. Trans.* 41 283–287. 10.1042/bst20120192 23356298

[B16] Chew J. R. J. Chuah S. J. Teo K. Y. W. Zhang S. Lai R. C. Fu J. H. (2019). Mesenchymal stem cell exosomes enhance periodontal ligament cell functions and promote periodontal regeneration. *Acta Biomater.* 89 252–264. 10.1016/j.actbio.2019.03.021 30878447

[B17] Cho B. S. Kim J. O. Ha D. H. Yi Y. W. (2018). Exosomes derived from human adipose tissue-derived mesenchymal stem cells alleviate atopic dermatitis. *Stem Cell Res. Ther.* 9:187. 10.1186/s13287-018-0939-5 29996938 PMC6042362

[B18] Colao I. L. Corteling R. Bracewell D. Wall I. (2018). Manufacturing exosomes: a promising therapeutic platform. *Trends Mol. Med.* 24 242–256. 10.1016/j.molmed.2018.01.006 29449149

[B19] Cui G. H. Guo H. D. Li H. Zhai Y. Gong Z. B. Wu J. (2019). RVG-modified exosomes derived from mesenchymal stem cells rescue memory deficits by regulating inflammatory responses in a mouse model of Alzheimer’s disease. *Immun. Ageing* 16:10. 10.1186/s12979-019-0150-2 31114624 PMC6515654

[B20] Dabrowska S. Andrzejewska A. Lukomska B. Janowski M. (2019). Neuroinflammation as a target for treatment of stroke using mesenchymal stem cells and extracellular vesicles. *J. Neuroinflammation* 16:178. 10.1186/s12974-019-1571-8 31514749 PMC6743114

[B21] Das M. Mayilsamy K. Mohapatra S. S. Mohapatra S. (2019). Mesenchymal stem cell therapy for the treatment of traumatic brain injury: progress and prospects. *Rev. Neurosci.* 30 839–855. 10.1515/revneuro-2019-0002 31203262

[B22] Doeppner T. R. Herz J. Gorgens A. Schlechter J. Ludwig A. K. Radtke S. (2015). Extracellular vesicles improve post-stroke neuroregeneration and prevent postischemic immunosuppression. *Stem Cells Transl. Med.* 4 1131–1143. 10.5966/sctm.2015-0078 26339036 PMC4572905

[B23] Ezquer F. Ezquer M. Contador D. Ricca M. Simon V. Conget P. (2012). The antidiabetic effect of mesenchymal stem cells is unrelated to their transdifferentiation potential but to their capability to restore Th1/Th2 balance and to modify the pancreatic microenvironment. *Stem Cells* 30 1664–1674. 10.1002/stem.1132 22644660

[B24] Fennema E. M. Tchang L. A. H. Yuan H. van Blitterswijk C. A. Martin I. Scherberich A. (2018). Ectopic bone formation by aggregated mesenchymal stem cells from bone marrow and adipose tissue: a comparative study. *J. Tissue Eng. Regen. Med.* 12 e150–e158. 10.1002/term.2453 28485099

[B25] Gao J. Dennis J. E. Muzic R. F. Lundberg M. Caplan A. I. (2001). The dynamic in vivo distribution of bone marrow-derived mesenchymal stem cells after infusion. *Cells Tissues Organs* 169 12–20. 10.1159/000047856 11340257

[B26] Gimble J. Guilak F. (2003). Adipose-derived adult stem cells: isolation, characterization, and differentiation potential. *Cytotherapy* 5 362–369. 10.1080/14653240310003026 14578098

[B27] Grange C. Tapparo M. Bruno S. Chatterjee D. Quesenberry P. J. Tetta C. (2014). Biodistribution of mesenchymal stem cell-derived extracellular vesicles in a model of acute kidney injury monitored by optical imaging. *Int. J. Mol. Med.* 33 1055–1063. 10.3892/ijmm.2014.1663 24573178 PMC4020482

[B28] György B. Szabó T. G. Pásztói M. Pál Z. Misják P. Aradi B. (2011). Membrane vesicles, current state-of-the-art: emerging role of extracellular vesicles. *Cell. Mol. Life Sci.* 68 2667–2688. 10.1007/s00018-011-0689-3 21560073 PMC3142546

[B29] Haraszti R. A. Miller R. Stoppato M. Sere Y. Y. Coles A. Didiot M.-C. (2018). Exosomes produced from 3D cultures of MSCs by tangential flow filtration show higher yield and improved activity. *Mol. Ther.* 26 2838–2847. 10.1016/j.ymthe.2018.09.015 30341012 PMC6277553

[B30] Heng B. C. Cao T. Lee E. H. (2004). Directing stem cell differentiation into the chondrogenic lineage *in vitro*. *Stem Cells* 22 1152–1167. 10.1634/stemcells.2004-0062 15579636

[B31] Herrera M. B. Bussolati B. Bruno S. Morando L. Mauriello-Romanazzi G. Sanavio F. (2007). Exogenous mesenchymal stem cells localize to the kidney by means of CD44 following acute tubular injury. *Kidney Int.* 72 430–441. 10.1038/sj.ki.5002334 17507906

[B32] Hsieh J. Y. Wang H. W. Chang S. J. Liao K. H. Lee I. H. Lin W. S. (2013). Mesenchymal stem cells from human umbilical cord express preferentially secreted factors related to neuroprotection, neurogenesis, and angiogenesis. *PLoS One* 8:e72604. 10.1371/journal.pone.0072604 23991127 PMC3749979

[B33] Jeong J. O. Han J. W. Kim J. M. Cho H. J. Park C. Lee N. (2011). Malignant tumor formation after transplantation of short-term cultured bone marrow mesenchymal stem cells in experimental myocardial infarction and diabetic neuropathy. *Circ. Res.* 108 1340–1347. 10.1161/circresaha.110.239848 21493893 PMC3109741

[B34] Karaoz E. Sun E. Demir C. S. (2019). Mesenchymal stem cell-derived exosomes do not promote the proliferation of cancer cells in vitro. *Int. J. Physiol. Pathophysiol. Pharmacol.* 11 177–189. 31523364 PMC6737426

[B35] Karimi N. Cvjetkovic A. Jang S. C. Crescitelli R. Hosseinpour Feizi M. A. Nieuwland R. (2018). Detailed analysis of the plasma extracellular vesicle proteome after separation from lipoproteins. *Cell. Mol. Life Sci.* 75 2873–2886. 10.1007/s00018-018-2773-4 29441425 PMC6021463

[B36] Katsuda T. Ochiya T. (2015). Molecular signatures of mesenchymal stem cell-derived extracellular vesicle-mediated tissue repair. *Stem Cell Res. Ther.* 6:212. 10.1186/s13287-015-0214-y 26560482 PMC4642616

[B37] Kim D. K. Nishida H. An S. Y. Shetty A. K. Bartosh T. J. Prockop D. J. (2016). Chromatographically isolated CD63+CD81+ extracellular vesicles from mesenchymal stromal cells rescue cognitive impairments after TBI. *Proc. Natl. Acad. Sci. U.S.A.* 113 170–175. 10.1073/pnas.1522297113 26699510 PMC4711859

[B38] Kim S. J. Moon G. J. Cho Y. H. Kang H. Y. Hyung N. K. Kim D. (2012). Circulating mesenchymal stem cells microparticles in patients with cerebrovascular disease. *PLoS One* 7:e37036. 10.1371/journal.pone.0037036 22615882 PMC3352849

[B39] Klyushnenkova E. Mosca J. D. Zernetkina V. Majumdar M. K. Beggs K. J. Simonetti D. W. (2005). T cell responses to allogeneic human mesenchymal stem cells: immunogenicity, tolerance, and suppression. *J. Biomed. Sci.* 12 47–57. 10.1007/s11373-004-8183-7 15864738

[B40] Kooijmans S. A. Aleza C. G. Roffler S. R. van Solinge W. W. Vader P. Schiffelers R. M. (2016). Display of GPI-anchored anti-EGFR nanobodies on extracellular vesicles promotes tumour cell targeting. *J. Extracell. Vesicles* 5:31053. 10.3402/jev.v5.31053 26979463 PMC4793259

[B41] Kordelas L. Rebmann V. Ludwig A. K. Radtke S. Ruesing J. Doeppner T. R. (2014). MSC-derived exosomes: a novel tool to treat therapy-refractory graft-versus-host disease. *Leukemia* 28 970–973. 10.1038/leu.2014.41 24445866

[B42] Kotmakçı M. Çetintaş V. (2015). Extracellular vesicles as natural nanosized delivery systems for small-molecule drugs and genetic material: steps towards the future nanomedicines. *J. Pharm. Pharm. Sci.* 18 396–413. 10.18433/j36w3x 26517135

[B43] Kuang M. J. Huang Y. Zhao X. G. Zhang R. Ma J. X. Wang D. C. (2019). Exosomes derived from Wharton’s jelly of human umbilical cord mesenchymal stem cells reduce osteocyte apoptosis in glucocorticoid-induced osteonecrosis of the femoral head in rats via the miR-21-PTEN-AKT signalling pathway. *Int. J. Biol. Sci.* 15 1861–1871. 10.7150/ijbs.32262 31523188 PMC6743291

[B44] Lai R. C. Arslan F. Lee M. M. Sze N. S. Choo A. Chen T. S. (2010). Exosome secreted by MSC reduces myocardial ischemia/reperfusion injury. *Stem Cell Res.* 4 214–222. 10.1016/j.scr.2009.12.003 20138817

[B45] Lee M. Ban J. J. Yang S. Im W. Kim M. (2018). The exosome of adipose-derived stem cells reduces beta-amyloid pathology and apoptosis of neuronal cells derived from the transgenic mouse model of Alzheimer’s disease. *Brain Res.* 1691 87–93. 10.1016/j.brainres.2018.03.034 29625119

[B46] Lee M. Liu T. Im W. Kim M. (2016). Exosomes from adipose-derived stem cells ameliorate phenotype of Huntington’s disease in vitro model. *Eur. J. Neurosci.* 44 2114–2119. 10.1111/ejn.13275 27177616

[B47] Lener T. Gimona M. Aigner L. Börger V. Buzas E. Camussi G. (2015). Applying extracellular vesicles based therapeutics in clinical trials – an ISEV position paper. *J. Extracell. Vesicles* 4 30087–30087. 10.3402/jev.v4.30087 26725829 PMC4698466

[B48] Li J. He X. Deng Y. Yang C. (2019). An update on isolation methods for proteomic studies of extracellular vesicles in biofluids. *Molecules* 24:3516. 10.3390/molecules24193516 31569778 PMC6803898

[B49] Li J. Lee Y. Johansson H. J. Mager I. Vader P. Nordin J. Z. (2015). Serum-free culture alters the quantity and protein composition of neuroblastoma-derived extracellular vesicles. *J. Extracell. Vesicles* 4:26883. 10.3402/jev.v4.26883 26022510 PMC4447833

[B50] Li T. Yan Y. Wang B. Qian H. Zhang X. Shen L. (2013). Exosomes derived from human umbilical cord mesenchymal stem cells alleviate liver fibrosis. *Stem Cells Dev.* 22 845–854. 10.1089/scd.2012.0395 23002959 PMC3585469

[B51] Lotvall J. Hill A. F. Hochberg F. Buzas E. I. Di Vizio D. Gardiner C. (2014). Minimal experimental requirements for definition of extracellular vesicles and their functions: a position statement from the international society for extracellular vesicles. *J. Extracell. Vesicles* 3:26913. 10.3402/jev.v3.26913 25536934 PMC4275645

[B52] McKee C. Chaudhry G. R. (2017). Advances and challenges in stem cell culture. *Colloids Surf. B Biointerfaces* 159 62–77. 10.1016/j.colsurfb.2017.07.051 28780462

[B53] Mendt M. Kamerkar S. Sugimoto H. McAndrews K. M. Wu C. C. Gagea M. (2018). Generation and testing of clinical-grade exosomes for pancreatic cancer. *JCI Insight* 3:e99263. 10.1172/jci.insight.99263 29669940 PMC5931131

[B54] Mennan C. Garcia J. Roberts S. Hulme C. Wright K. (2019). A comprehensive characterisation of large-scale expanded human bone marrow and umbilical cord mesenchymal stem cells. *Stem Cell Res. Ther.* 10:99. 10.1186/s13287-019-1202-4 30885254 PMC6421680

[B55] Mittelbrunn M. Sanchez-Madrid F. (2012). Intercellular communication: diverse structures for exchange of genetic information. *Nat. Rev. Mol. Cell Biol.* 13 328–335. 10.1038/nrm3335 22510790 PMC3738855

[B56] Mol E. A. Goumans M. J. Doevendans P. A. Sluijter J. P. G. Vader P. (2017). Higher functionality of extracellular vesicles isolated using size-exclusion chromatography compared to ultracentrifugation. *Nanomedicine* 13 2061–2065. 10.1016/j.nano.2017.03.011 28365418

[B57] Monguio-Tortajada M. Galvez-Monton C. Bayes-Genis A. Roura S. Borras F. E. (2019). Extracellular vesicle isolation methods: rising impact of size-exclusion chromatography. *Cell. Mol. Life Sci.* 76 2369–2382. 10.1007/s00018-019-03071-y 30891621 PMC11105396

[B58] Moon G. J. Sung J. H. Kim D. H. Kim E. H. Cho Y. H. Son J. P. (2019). Application of mesenchymal stem cell-derived extracellular vesicles for stroke: biodistribution and microRNA study. *Transl. Stroke Res.* 10 509–521. 10.1007/s12975-018-0668-1 30341718

[B59] Nassar W. El-Ansary M. Sabry D. Mostafa M. A. Fayad T. Kotb E. (2016). Umbilical cord mesenchymal stem cells derived extracellular vesicles can safely ameliorate the progression of chronic kidney diseases. *Biomater. Res.* 20:21. 10.1186/s40824-016-0068-0 27499886 PMC4974791

[B60] Natasha G. Gundogan B. Tan A. Farhatnia Y. Wu W. Rajadas J. (2014). Exosomes as immunotheranostic nanoparticles. *Clin. Ther.* 36 820–829. 10.1016/j.clinthera.2014.04.019 24863261

[B61] Nawaz M. Fatima F. Vallabhaneni K. C. Penfornis P. Valadi H. Ekstrom K. (2016). Extracellular vesicles: evolving factors in stem cell biology. *Stem Cells Int.* 2016:1073140. 10.1155/2016/1073140 26649044 PMC4663346

[B62] Nekanti U. Mohanty L. Venugopal P. Balasubramanian S. Totey S. Ta M. (2010). Optimization and scale-up of Wharton’s jelly-derived mesenchymal stem cells for clinical applications. *Stem Cell Res.* 5 244–254. 10.1016/j.scr.2010.08.005 20880767

[B63] Ni H. Yang S. Siaw-Debrah F. Hu J. Wu K. He Z. (2019). Exosomes derived from bone mesenchymal stem cells ameliorate early inflammatory responses following traumatic brain injury. *Front. Neurosci.* 13:14. 10.3389/fnins.2019.00014 30733666 PMC6354067

[B64] Nordin J. Z. Lee Y. Vader P. Mager I. Johansson H. J. Heusermann W. (2015). Ultrafiltration with size-exclusion liquid chromatography for high yield isolation of extracellular vesicles preserving intact biophysical and functional properties. *Nanomedicine* 11 879–883. 10.1016/j.nano.2015.01.003 25659648

[B65] Nouraee N. Mowla S. J. (2015). miRNA therapeutics in cardiovascular diseases: promises and problems. *Front. Genet.* 6:232. 10.3389/fgene.2015.00232 26175755 PMC4485214

[B66] Oliver-Vila I. Coca M. I. Grau-Vorster M. Pujals-Fonts N. Caminal M. Casamayor-Genesca A. (2016). Evaluation of a cell-banking strategy for the production of clinical grade mesenchymal stromal cells from Wharton’s jelly. *Cytotherapy* 18 25–35. 10.1016/j.jcyt.2015.10.001 26549383

[B67] Ophelders D. R. Wolfs T. G. Jellema R. K. Zwanenburg A. Andriessen P. Delhaas T. (2016). Mesenchymal stromal cell-derived extracellular vesicles protect the fetal brain after hypoxia-ischemia. *Stem Cells Transl. Med.* 5 754–763. 10.5966/sctm.2015-0197 27160705 PMC4878333

[B68] Pachler K. Lener T. Streif D. Dunai Z. A. Desgeorges A. Feichtner M. (2017). A good manufacturing practice-grade standard protocol for exclusively human mesenchymal stromal cell-derived extracellular vesicles. *Cytotherapy* 19 458–472. 10.1016/j.jcyt.2017.01.001 28188071

[B69] Phan J. Kumar P. Hao D. Gao K. Farmer D. Wang A. (2018). Engineering mesenchymal stem cells to improve their exosome efficacy and yield for cell-free therapy. *J. Extracell. Vesicles* 7:1522236. 10.1080/20013078.2018.1522236 30275938 PMC6161586

[B70] Pitt J. M. André F. Amigorena S. Soria J. C. Eggermont A. Kroemer G. (2016). Dendritic cell-derived exosomes for cancer therapy. *J. Clin. Invest.* 126 1224–1232. 10.1172/JCI81137 27035813 PMC4811123

[B71] Rao J. Dragulescu-Andrasi A. Yao H. (2007). Fluorescence imaging in vivo: recent advances. *Curr. Opin. Biotechnol.* 18 17–25. 10.1016/j.copbio.2007.01.003 17234399

[B72] Roura S. Pujal J. M. Gálvez-Montón C. Bayes-Genis A. (2015). The role and potential of umbilical cord blood in an era of new therapies: a review. *Stem Cell Res. Ther.* 6:123. 10.1186/s13287-015-0113-2 26133757 PMC4489204

[B73] Shao L. Zhang Y. Lan B. Wang J. Zhang Z. Zhang L. (2017). MiRNA-sequence indicates that mesenchymal stem cells and exosomes have similar mechanism to enhance cardiac repair. *Biomed. Res. Int.* 2017:4150705. 10.1155/2017/4150705 28203568 PMC5292186

[B74] Shelke G. V. Lasser C. Gho Y. S. Lotvall J. (2014). Importance of exosome depletion protocols to eliminate functional and RNA-containing extracellular vesicles from fetal bovine serum. *J. Extracell. Vesicles* 3:24783. 10.3402/jev.v3.24783 25317276 PMC4185091

[B75] Stranska R. Gysbrechts L. Wouters J. Vermeersch P. Bloch K. Dierickx D. (2018). Comparison of membrane affinity-based method with size-exclusion chromatography for isolation of exosome-like vesicles from human plasma. *J. Transl. Med.* 16:1. 10.1186/s12967-017-1374-6 29316942 PMC5761138

[B76] Tetta C. Ghigo E. Silengo L. Deregibus M. C. Camussi G. (2013). Extracellular vesicles as an emerging mechanism of cell-to-cell communication. *Endocrine* 44 11–19. 10.1007/s12020-012-9839-0 23203002 PMC3726927

[B77] Thomi G. Surbek D. Haesler V. Joerger-Messerli M. Schoeberlein A. (2019). Exosomes derived from umbilical cord mesenchymal stem cells reduce microglia-mediated neuroinflammation in perinatal brain injury. *Stem Cell Res. Ther.* 10:105. 10.1186/s13287-019-1207-z 30898154 PMC6429800

[B78] Timmins N. E. Kiel M. Günther M. Heazlewood C. Doran M. R. Brooke G. (2012). Closed system isolation and scalable expansion of human placental mesenchymal stem cells. *Biotechnol. Bioeng.* 109 1817–1826. 10.1002/bit.24425 22249999

[B79] Tögel F. Yang Y. Zhang P. Hu Z. Westenfelder C. (2008). Bioluminescence imaging to monitor the in vivo distribution of administered mesenchymal stem cells in acute kidney injury. *Am. J. Physiol. Renal Physiol.* 295 F315–F321. 10.1152/ajprenal.00098.2008 18480180 PMC4063418

[B80] Ullah I. Subbarao R. B. Rho G. J. (2015). Human mesenchymal stem cells – current trends and future prospective. *Biosci. Rep.* 35:e00191. 10.1042/BSR20150025 25797907 PMC4413017

[B81] Vakhshiteh F. Atyabi F. Ostad S. N. (2019). Mesenchymal stem cell exosomes: a two-edged sword in cancer therapy. *Int. J. Nanomed.* 14 2847–2859. 10.2147/IJN.S200036 31114198 PMC6488158

[B82] Valadi H. Ekstrom K. Bossios A. Sjostrand M. Lee J. J. Lotvall J. O. (2007). Exosome-mediated transfer of mRNAs and microRNAs is a novel mechanism of genetic exchange between cells. *Nat. Cell Biol.* 9 654–659. 10.1038/ncb1596 17486113

[B83] Wang D. Gao B. Yue J. Liu F. Liu Y. Fu W. (2019). Exosomes from mesenchymal stem cells expressing miR-125b inhibit neointimal hyperplasia via myosin IE. *J. Cell. Mol. Med.* 23 1528–1540. 10.1111/jcmm.14060 30484954 PMC6349157

[B84] Wang J. H. Forterre A. V. Zhao J. Frimannsson D. O. Delcayre A. Antes T. J. (2018). Anti-HER2 scFv-directed extracellular vesicle-mediated mRNA-based gene delivery inhibits growth of HER2-positive human breast tumor xenografts by prodrug activation. *Mol. Cancer Ther.* 17 1133–1142. 10.1158/1535-7163.MCT-17-0827 29483213 PMC5932266

[B85] Wang S. Guo L. Ge J. Yu L. Cai T. Tian R. (2015). Excess integrins cause lung entrapment of mesenchymal stem cells. *Stem Cells* 33 3315–3326. 10.1002/stem.2087 26148841

[B86] Wen S. Dooner M. Papa E. Del Tatto M. Pereira M. Borgovan T. (2019). Biodistribution of mesenchymal stem cell-derived extracellular vesicles in a radiation injury bone marrow murine model. *Int. J. Mol. Sci.* 20:5468. 10.3390/ijms20215468 31684046 PMC6861905

[B87] Whitford W. Guterstam P. (2019). Exosome manufacturing status. *Future Med. Chem.* 11 1225–1236. 10.4155/fmc-2018-0417 31280675

[B88] Whitford W. Ludlow J. Cadwel J. (2015). Continuous production of exosomes. *Genet. Eng. Biotechnol. News* 35:34 10.1089/gen.35.16.15

[B89] Witwer K. W. Buzas E. I Bemis L. T. Bora A. Lasser C. Lotvall J. (2013). Standardization of sample collection, isolation and analysis methods in extracellular vesicle research. *J. Extracell. Vesicles* 2:20360. 10.3402/jev.v2i0.20360 24009894 PMC3760646

[B90] Xin H. Li Y. Cui Y. Yang J. J. Zhang Z. G. Chopp M. (2013a). Systemic administration of exosomes released from mesenchymal stromal cells promote functional recovery and neurovascular plasticity after stroke in rats. *J. Cereb. Blood Flow Metab.* 33 1711–1715. 10.1038/jcbfm.2013.152 23963371 PMC3824189

[B91] Xin H. Li Y. Liu Z. Wang X. Shang X. Cui Y. (2013b). MiR-133b promotes neural plasticity and functional recovery after treatment of stroke with multipotent mesenchymal stromal cells in rats via transfer of exosome-enriched extracellular particles. *Stem Cells* 31 2737–2746. 10.1002/stem.1409 23630198 PMC3788061

[B92] Yagi H. Soto-Gutierrez A. Parekkadan B. Kitagawa Y. Tompkins R. G. Kobayashi N. (2010). Mesenchymal stem cells: mechanisms of immunomodulation and homing. *Cell Transplant.* 19 667–679. 10.3727/096368910X508762 20525442 PMC2957533

[B93] Yáñez-Mó M. Siljander P. R. Andreu Z. Zavec A. B. Borràs F. E. Buzas E. I. (2015). Biological properties of extracellular vesicles and their physiological functions. *J. Extracell. Vesicles* 4:27066. 10.3402/jev.v4.27066 25979354 PMC4433489

[B94] Yaoxiang S. Hui S. Siqi Y. Cheng J. Xu Z. Bin Z. (2018). Human mesenchymal stem cell derived exosomes alleviate type 2 diabetes mellitus by reversing peripheral insulin resistance and relieving β-cell destruction. *ACS Nano* 12 7613–7628. 10.1021/acsnano.7b07643 30052036

[B95] Yeo R. W. Y. Lai R. C. Zhang B. Tan S. S. Yin Y. Teh B. J. (2013). Mesenchymal stem cell: an efficient mass producer of exosomes for drug delivery. *Adv. Drug Deliv. Rev.* 65 336–341. 10.1016/j.addr.2012.07.001 22780955

[B96] Yin K. Wang S. Zhao R. C. (2019). Exosomes from mesenchymal stem/stromal cells: a new therapeutic paradigm. *Biomark. Res.* 7:8. 10.1186/s40364-019-0159-x 30992990 PMC6450000

[B97] Zhang J. Guan J. Niu X. Hu G. Guo S. Li Q. (2015). Exosomes released from human induced pluripotent stem cells-derived MSCs facilitate cutaneous wound healing by promoting collagen synthesis and angiogenesis. *J. Transl. Med.* 13:49. 10.1186/s12967-015-0417-0 25638205 PMC4371881

[B98] Zhang Y. Liu Y. Liu H. Tang W. H. (2019). Exosomes: biogenesis, biologic function and clinical potential. *Cell Biosci.* 9:19. 10.1186/s13578-019-0282-2 30815248 PMC6377728

[B99] Zhang Z.-Y. Teoh S.-H. Hui J. H. Fisk N. M. Choolani M. Chan J. K. (2012). The potential of human fetal mesenchymal stem cells for off-the-shelf bone tissue engineering application. *Biomaterials* 33 2656–2672. 10.1016/j.biomaterials.2011.12.025 22217806

[B100] Zhao Y. Jiang Z. Zhao T. Ye M. Hu C. Yin Z. (2012). Reversal of type 1 diabetes via islet beta cell regeneration following immune modulation by cord blood-derived multipotent stem cells. *BMC Med.* 10:3. 10.1186/1741-7015-10-3 22233865 PMC3322343

[B101] Zhou Y. Xu H. Xu W. Wang B. Wu H. Tao Y. (2013). Exosomes released by human umbilical cord mesenchymal stem cells protect against cisplatin-induced renal oxidative stress and apoptosis in vivo and in vitro. *Stem Cell Res. Ther.* 4:34. 10.1186/scrt194 23618405 PMC3707035

[B102] Zhuang X. Xiang X. Grizzle W. Sun D. Zhang S. Axtell R. C. (2011). Treatment of brain inflammatory diseases by delivering exosome encapsulated anti-inflammatory drugs from the nasal region to the brain. *Mol. Ther.* 19 1769–1779. 10.1038/mt.2011.164 21915101 PMC3188748

